# Developing translational medicine professionals: the Marie Skłodowska-Curie action model

**DOI:** 10.1186/s12967-016-1088-1

**Published:** 2016-11-29

**Authors:** Alessandra Petrelli, Berent J. Prakken, Norman D. Rosenblum, EUtrain fellows

**Affiliations:** 1 Laboratory of Translational Immunology, Wilhelmina Children’s Hospital, University Medical Center of Utrecht (UMCU), Lundlaan 6, 3584 EA Utrecht, Netherlands; 2 Diabetes Research Institute, San Raffaele Vita-Salute University, Milan, Italy; 3 The Hospital for Sick Children, Peter Gilgan Centre for Research and Learning, University of Toronto, Toronto, ON Canada

## Abstract

End goal of translational medicine is to combine disciplines and expertise to eventually promote improvement of the global healthcare system by delivering effective therapies to individuals and society. Well-trained experts of the translational medicine process endowed with profound knowledge of biomedical technology, ethical and clinical issues, as well as leadership and teamwork abilities are essential for the effective development of tangible therapeutic products for patients. In this article we focus on education and, in particular, we discuss how programs providing training on the broad spectrum of the translational medicine continuum have still a limited degree of diffusion and do not provide professional support and mentorship in the long-term, resulting in the lack of well established professionals of translational medicine (TMPs) in the scientific community. Here, we describe the Marie Skłodowska-Curie Actions program ITN-EUtrain (EUropean Translational tRaining for Autoimmunity & Immune manipulation Network) where training on the Translational Medicine machinery was integrated with education on professional and personal skills, mentoring, and a long-lasting network of TMPs.

## The need for translational medicine professionals (TMPs)

The twentieth century has been revolutionary in terms of biomedical discoveries and innovations in technologies, e.g. high-throughput screenings and omics. The large amount of knowledge so derived has great potential to prolong human life and enhance its quality. However, there is considerable concern of an insufficient translation of this knowledge into tangible products with clear clinical impact [1]. This gap in the translational process is described as the *valley of death.* The valley represents the challenges that clinicians and scientists are facing in the process of developing effective therapies for patients, resulting in only a small fraction of all efficacious pharmacological agents in preclinical models being licenced after Phase III clinical testing [2]. Possible explanations are: (1) animal models do not correctly represent human diseases due to significant differences in biology [3], (2) overestimation of drug effects in preclinical models due to the higher probability of positive results to be published [4], (3) limited well-designed clinical trials in the era of precision medicine [5] and (4) irreproducibility of scientific findings [6], which is only to a small extent caused by scientific misconduct [7]. Despite translational medicine is a priority for the scientific community, professional figures specifically trained to facilitate the complex processes of the translational medicine continuum remain scarce. Translation requires a bi-directional effort starting from the identification of clinical needs and ending with the development of technology/products to be brought back into the clinic. Lack of translation results in waste of public money and resources, delay in scientific progress, and therefore, loss of potential benefits for human health. One proposed solution to bridge the gap between research and clinical care is the use of an educational model based on training and professional growth to foster the necessary skills required for the development of experts of the translational medicine process (i.e. TMPs). TMPs are expert in the translational medicine itinerary and are equipped to act as interfaces supporting efficient communication among different parties of the process, e.g. clinicians, regulatory affairs, companies, basic researchers.

## Need for an educational shift in the training of young professionals

Dynamic change within biomedical research and healthcare requires a synchronous development of learning environments and opportunities to enhance the knowledge and skills of young professionals. Just like the acknowledged need for a shift in the general educational system gave rise to the P21 [8], a collaborative partnership among education, business, community and government leaders, the accelerated rate of changes in translational medicine requires a similar collaborative effort. The aims should be to train and support a community of TMPs to deal with the need of producing tangible products for the treatment of human diseases. Despite there being many excellent scientists and clinicians as well as experts of regulatory affairs and industry, professionals that can oversee the entire translational process as well as shared platforms where those experts can easily connect and access each other’s information and skills are still scarce.

What educational innovations are required to positively impact on the translational process? First, there is the need to set up educational programs fostering the development of TMPs with multidimensional skills (ranging from specific competences on the translational process to communication, coaching, creative thinking, problem solving, management). Secondly, TMPs should avoid the “silo” mentality, a narrow outlook that has contributed to low efficiency and failure in science, but rather break down barriers and cross boundaries by sharing information and knowledge with other scientists, healthcare providers and industry [9].

## Existing educational programs in translational medicine

Educational programs providing training, at different levels, related to stages of the therapeutic development pipeline, and addressing the needs of individuals with distinct roles in translational research (i.e. government, industry and academia) are available. Examples are the clinical translational science (CTS) programs developed in the USA for graduate and postgraduate students [10], and the Canadian Child Health Clinician Scientist Program (CCHCSP), providing training for clinical researchers to develop knowledge and skills for a career as an independent scientist in child health research [11]. These programs support the development of the translational scientist identity by offering a comprehensive training on both ‘foundational’ skills, such as research methodologies and data management, and ‘functional’ skills, such as communication, ethics, leadership and teamwork [12]. However, these programs have a limited degree of diffusion and are not structured to provide peer support and mentorship in the long-term. The latter point, in our opinion, is an essential requirement to sustain group identity, and provide professional guidance as well as concrete help to access information or other types of resources related to translational medicine.

Another approach to cross the *valley of death* is to develop a community of TMPs with different skills and expertise that can be shared and utilized by all members. The Eureka Institute [13] founded in 2007, is an example of such a community. Currently, it comprises a worldwide network of over 200 fellows trained in interdisciplinary teamwork, open-minded thinking and principles of translational medicine (e.g. biology, intellectual property, funding, regulation and trial design) during a week-long course held in Siracuse (Sicily, Italy). Teaching comprises interactive sessions led by experts in the process of translational research, including scientific directors and deans of academic institutes, editors of high-impact journals, industry, and representatives of patient organizations. Fellows are trained in leadership, teamwork and communication to different types of audience as well as ethical and technical principles of the translational medicine machinery. To maximize the learning experience, the training technique requires students to work together to design strategies and solutions to real-life issues previously encountered by experienced TMPs. This network of translational researchers is growing, meetings and seminars are routinely scheduled, news in the translational medicine field are shared on social networks and a database is currently under development including participants expertise and tools as well as a section to share ideas for the development of joint projects. The challenge now lies in its long-term sustainability eventually providing TMPs access to continuously updated knowledge and tools.

## The Marie Skłodowska-Curie ITN model

European Union Marie Skłodowska-Curie Actions, named after the double Nobel Prize winning scientist for her work on radioactivity, support researchers working across all disciplines, including industrial and academic research studies, giving the researcher the possibility to gain experience abroad and develop competences useful to enhance future careers. This program provides a remarkable tool, the innovative training network (ITN), allowing early-stage researchers to receive doctoral training and develop transferable skills by working on joint research projects within a worldwide network. One such ITN, EUtrain (EUropean Translational tRaining for Autoimmunity & Immune manipulation Network, http://www.eutrain-network.eu), was funded in 2011 and aimed at training a pool of young investigators in Translational Medicine providing (1) education on professional and personal skills, (2) mentoring, and (3) long-lasting network of TMPs.

EUtrain focused on patient-centred research with respect to diagnosis, prognosis and/or treatment of immune-mediated diseases. Participating fellows had diverse backgrounds (i.e. basic scientists, biotechnologists, medical doctors, chemists and engineers) and conducted 3-year research training in one of the 10 participating academic institutes based in the Netherlands, Italy, Germany, United Kingdom and France, or companies (Esaote Spa, Italy; Cavadis BV, The Netherlands; Proteros Biostructures GmbH, Germany). Identification of clinical needs provided the basis to motivate generation of translational solutions. EUtrain partners performed joint research projects combining basic immune biology studies on the regulation of inflammation with the development of novel high throughput and imaging technologies to detect biological markers of disease, and integrate this with clinical studies and systems biology. As an example, Work Package (WP) 2 aimed to identify both conventional and innovative biological markers for monitoring disease activity, progression, response to therapy and prognosis. WP2 included 4 joint research projects held by 4 early-stage researchers located in academia and industry, consisting of (1) develop and validate commercial assays for quantification of biomarkers of inflammation, (2) perform a global proteomic screening for novel protein biomarkers in blood of children with inflammatory diseases, (3) analyze the bias towards tolerance or inflammation based on functional and molecular analysis of the relevant pathways, and (4) develop a biomarker set that may predict the long-term risk of cardiovascular complications.

Besides the Eureka experience, trainees received additional training in specific aspects of the translational process from the conception of an idea to clinical testing and, ultimately, the development of a treatment for patients. Additionally, EUtrain fellows were coached on professional skills, such as communication to a scientific and layman audience, collaboration, conflict solving, teamwork, and mentor/mentee relationships. EUtrain fellows experienced network-wide training events, such as the grant writing weekend, workshops on presentation skills, effective communication, moderation of scientific meetings, valorisation of results and intellectual property (IP).

Core of the training was mentorship on career and personal development, which was performed on a regular basis by experienced TMPs. Each career step requires the identification of an experienced and knowledgeable figure providing guidance and advice on work, career, or professional development (i.e. mentor); on several occasions during the training it was raised awareness on the importance of the identification of a mentor to fulfill the needs of one’s life and career goals. Fellows met regularly in informal settings and were repeatedly exposed to these sessions and principles at different occasions during the 3-year training.

Here we provide a summary of the themes that emerged from the survey proposed to EUtrain fellows to determine whether and how EUtrain helped to achieve their professional goals. All survey respondents (13/14) agreed that the program enhanced their basic and translational knowledge, and enjoyed the integrated experience where network and training merged, allowing both scientific and personal growth. In particular, they all agreed that EUtrain helped creating a solid and long-lasting European-wide professional network as well as developing multidimensional skills. Thanks to EUtrain, fellows improved their technical competences and used them to answer clinical questions, got insights into the clinical practice, understood dynamics and objectives of academic and industrial research. Despite the overall achievement of goals, one fellow would have liked to have more time for training in personal and professional development, which was instead incompatible with lab duties. Notably, most of the fellows would not consider themselves independent researchers or TMPs yet at the end of the training, which might be due to their early career stage. It would be of interest to follow-up the experiences of these individuals, and to explore the effects of a similar type of training on more senior investigators. It is impossible to evaluate at this stage whether this training program will significantly improve the future development of clinically useful products. However, all EUtrain fellows have acquired the essential tools to be able to contribute to this goal. Future studies need to examine career outcomes to identity program components that effectively equip trainees with the skills needed to thrive in a translational environment.

## Conclusions

Fragmented expertise in the translational research process and lack of platforms designed to share knowledge and tools among professionals are some of the barriers to the development of tangible products with clinical impact. Only specifically trained professionals such as TMPs can facilitate research to cross the *valley of death*. The proposed approach to fill the existing gap in translational medicine consists in the integration of education within a network of professionals, allowing the sharing of skills and promoting collaborative efforts. This would also allow TMPs to access more or newer research tools and keep up to date on cutting-edge technologies and information provided by a large network. EUtrain fellows are now part of an international network (i.e. Eureka) from which they can obtain interdisciplinary and intersectoral support as well as identify a mentor for career guidance to ultimately attain their translational purposes. To close the translational gap there is urgent need of multidimensional TMPs endowed with creative and open-minded thinking, able to work in cross-functional teams, and embedded in a solid community of peers providing career guidance, theoretical framework and technical support.
